# Clinical presentations, laboratory and radiological findings, and treatments for 11,028 COVID-19 patients: a systematic review and meta-analysis

**DOI:** 10.1038/s41598-020-74988-9

**Published:** 2020-11-13

**Authors:** Carlos K. H. Wong, Janet Y. H. Wong, Eric H. M. Tang, C. H. Au, Abraham K. C. Wai

**Affiliations:** 1 Department of Family Medicine and Primary Care, Li Ka Shing Faculty of Medicine, The University of Hong Kong, Hong Kong, China; 2 Department of Pharmacology and Pharmacy, Li Ka Shing Faculty of Medicine, The University of Hong Kong, Hong Kong, China; 3 School of Nursing, Li Ka Shing Faculty of Medicine, The University of Hong Kong, Hong Kong, China; 4 Emergency Medicine Unit, Li Ka Shing, Faculty of Medicine, The University of Hong Kong, Hong Kong, China

**Keywords:** Microbiology, Diseases, Health care, Medical research, Risk factors

## Abstract

This systematic review and meta-analysis investigated the comorbidities, symptoms, clinical characteristics and treatment of COVID-19 patients. Epidemiological studies published in 2020 (from January–March) on the clinical presentation, laboratory findings and treatments of COVID-19 patients were identified from PubMed/MEDLINE and Embase databases. Studies published in English by 27th March, 2020 with original data were included. Primary outcomes included comorbidities of COVID-19 patients, their symptoms presented on hospital admission, laboratory results, radiological outcomes, and pharmacological and in-patient treatments. 76 studies were included in this meta-analysis, accounting for a total of 11,028 COVID-19 patients in multiple countries. A random-effects model was used to aggregate estimates across eligible studies and produce meta-analytic estimates. The most common comorbidities were hypertension (18.1%, 95% CI 15.4–20.8%). The most frequently identified symptoms were fever (72.4%, 95% CI 67.2–77.7%) and cough (55.5%, 95% CI 50.7–60.3%). For pharmacological treatment, 63.9% (95% CI 52.5–75.3%), 62.4% (95% CI 47.9–76.8%) and 29.7% (95% CI 21.8–37.6%) of patients were given antibiotics, antiviral, and corticosteroid, respectively. Notably, 62.6% (95% CI 39.9–85.4%) and 20.2% (95% CI 14.6–25.9%) of in-patients received oxygen therapy and non-invasive mechanical ventilation, respectively. This meta-analysis informed healthcare providers about the timely status of characteristics and treatments of COVID-19 patients across different countries.

PROSPERO Registration Number: CRD42020176589

## Introduction

Following the possible patient zero of coronavirus infection identified in early December 2019^1^, the Coronavirus Disease 2019 (COVID-19) has been recognized as a pandemic in mid-March 2020^2^, after the increasing global attention to the exponential growth of confirmed cases^3^. As on 29th March, 2020, around 690 thousand persons were confirmed infected, affecting 199 countries and territories around the world, in addition to 2 international conveyances: the Diamond Princess cruise ship harbored in Yokohama, Japan, and the Holland America's MS Zaandam cruise ship. Overall, more than 32 thousand died and about 146 thousand have recovered^4^.

A novel bat-origin virus, 2019 novel coronavirus, was identified by means of deep sequencing analysis. SARS-CoV-2 was closely related (with 88% identity) to two bat-derived severe acute respiratory syndrome (SARS)-like coronaviruses, bat-SL-CoVZC45 and bat-SL-CoVZXC21, but were more distant from SARS-CoV (about 79%) and MERS-CoV (about 50%)^5^, both of which were respectively responsible for two zoonotic human coronavirus epidemics in the early twenty-first century. Following a few initial human infections^6^, the disease could easily be transmitted to a substantial number of individuals with increased social gathering^7^ and population mobility during holidays in December and January^8^. An early report has described its high infectivity^9^ even before the infected becomes symptomatic^10^. These natural and social factors have potentially influenced the general progression and trajectory of the COVID-19 epidemiology.

By the end of March 2020, there have been approximately 3000 reports about COVID-19^11^. The number of COVID-19-related reports keeps growing everyday, yet it is still far from a clear picture on the spectrum of clinical conditions, transmissibility and mortality, alongside the limitation of medical reports associated with reporting in real time the evolution of an emerging pathogen in its early phase. Previous reports covered mostly the COVID-19 patients in China. With the spread of the virus to other continents, there is an imminent need to review the current knowledge on the clinical features and outcomes of the early patients, so that further research and measures on epidemic control could be developed in this epoch of the pandemic.

## Methods

### Search strategy and selection criteria

The systematic review was conducted according to the protocol registered in the PROSPERO database (CRD42020176589). Following the Preferred Reporting Items for Systematic Reviews and Meta-Analysis (PRISMA) guideline throughout this review, data were identified by searches of MEDLINE, Embase and references from relevant articles using the search terms "COVID", “SARS-CoV-2”, and “novel coronavirus” (Supplementary material 1). Articles published in English up to 27th March, 2020 were included. National containment measures have been implemented at many countries, irrespective of lockdown, curfew, or stay-at-home orders, since the mid of March 2020^12^, except for China where imposed Hubei province lockdown at 23th January 2020, Studies with original data including original articles, short and brief communication, letters, correspondences were included. Editorials, viewpoints, infographics, commentaries, reviews, or studies without original data were excluded. Studies were also excluded if they were animal studies, modelling studies, or did not measure symptoms presentation, laboratory findings, treatment and therapeutics during hospitalization.

After the removal of duplicate records, two reviewers (CW and CHA) independently screened the eligibility criteria of study titles, abstracts and full-texts, and reference lists of the studies retrieved by the literature search. Disagreements regarding the procedures of database search, study selection and eligibility were resolved by discussion. The second and the last authors (JW and AW) verified the eligibility of included studies.

### Outcomes definitions

Signs and symptoms were defined as the presentation of fever, cough, sore throat, headache, dyspnea, muscle pain, diarrhea, rhinorrhea, anosmia, and ageusia at the hospital admission^13^.

Laboratory findings included a complete blood count (white blood count, neutrophil, lymphocyte, platelet count), procalcitonin, prothrombin time, urea, and serum biochemical measurements (including electrolytes, renal-function and liver-function values, creatine kinase, lactate dehydrogenase, C-reactive protein, Erythrocyte sedimentation rate), and treatment measures (i.e. antiviral therapy, antibiotics, corticosteroid therapy, mechanical ventilation, intubation, respiratory support, and renal replacement therapy). Radiological outcomes included bilateral involvement identified and pneumonia identified by chest radiograph.

Comorbidities of patients evaluated in this study were hypertension, diabetes, chronic obstructive pulmonary disease (COPD), cardiovascular disease, chronic kidney disease, liver disease and cancer.

In-patient treatment included intensive care unit admission, oxygen therapy, non-invasive ventilation, mechanical ventilation, Extracorporeal membrane oxygenation (ECMO), renal replacement therapy, and pharmacological treatment. Use of antiviral and interferon drugs (Lopinavir/ritonavir, Ribavirin, Umifenovir, Interferon-alpha, or Interferon-beta), antibiotic drugs, corticosteroid, and inotropes (Nor-adrenaline, Adrenaline, Vasopressin, Phenylephrine, Dopamine, or Dobutamine) were considered.

### Data analysis

Three authors (CW, EHMT and CHA) extracted data using a standardized spreadsheet to record the article type, country of origin, surname of first author, year of publications, sample size, demographics, comorbidities, symptoms, laboratory and radiology results, pharmacological and non-pharmacological treatments.

We aggregated estimates across 90 eligible studies to produce meta-analytic estimates using a random-effects model. For dichotomous outcomes, we estimated the proportion and its respective 95% confidence interval. For laboratory parameters as continuous outcomes, we estimated the mean and standard deviation from the median and interquartile range if the mean and standard deviation were not available from the study^14^, and calculated the mean and its respective 95% confidence intervals. Random-effect models on DerSimonian and Laird method were adopted due to the significant heterogeneity, checked by the I^2^ statistics and the *p* values. I^2^ statistic of < 25%, 25–75% and ≥ 75% is considered as low, moderate, high likelihood of heterogeneity. Pooled estimates were calculated and presented by using forest plots. Publication bias was estimated by Egger’s regression test. Funnel plots of outcomes were also presented to assess publication bias.

All statistical analyses were conducted using the STATA Version 13.0 (Statacorp, College Station, TX). The random effects model was generated by the Stata packages ‘Metaprop’ for proportions^15^ and ‘Metan’ for continuous variables^16^.

## Results

**Figure 1:**
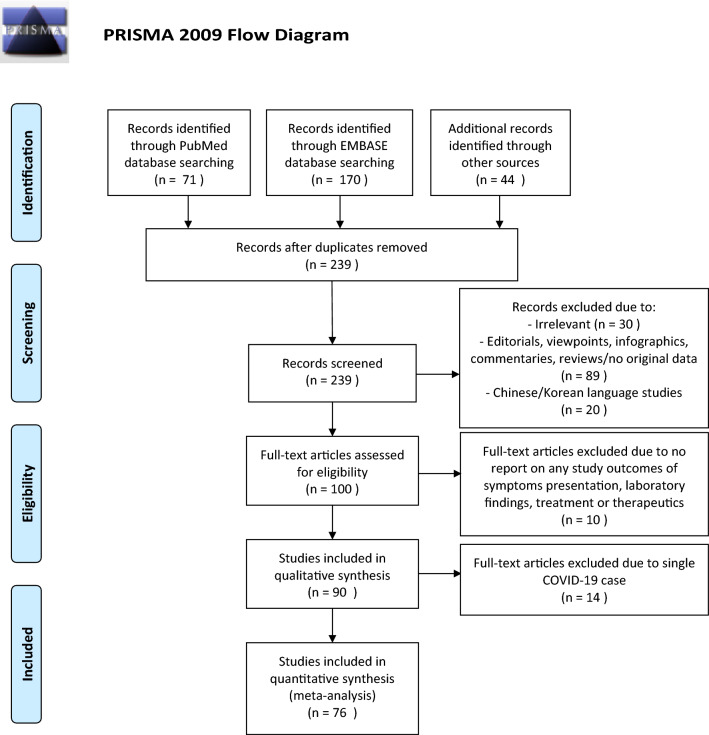
PRISMA flowchart reporting identification, searching and selection processes.

The selection and screen process are presented in Fig. 1. A total of 241 studies were found by our searching strategy (71 in PubMed and 170 in Embase). 46 records were excluded due to duplication. After screening the abstracts and titles, 100 English studies were with original data and included in full-text screening. By further excluding 10 studies with not reporting symptoms presentation, laboratory findings, treatment and therapeutics, 90 studies^17–106^ and 76 studies with more than one COVID-19 case^17–31,34–39,42–45,49–51,53,57–64,67,69,70,72–79,81–96,98,100–105^ were included in the current systematic review and meta-analysis respectively. 73.3%^66^ studies were conducted in China. Newcastle–Ottawa Quality Assessment Scale has been used to assess study quality of each included cohort study^107^. 30% (27/90) of included studies had satisfactory or good quality. The summary of the included study is shown in Table 1.

**Table 1:** Summary of 90 reviewed studies.

Study	Region/country	State/city	Hospital	Period of confirmed cases	N	Mean age (SD) (year)	Male (%)	Severe (%)
Xu et al.^17^	China	Guangzhou city	Guangzhou Eighth People’s Hospital	23 Jan 2020—4 Feb 2020	90	51.3 (NA)	43.3%	NA
Cao et al.^18^	China	Wuhan city	Zhongnan Hospital	3 Jan 2020–1 Feb 2020	102	52.7 (22.6)	52.0%	NA
Xiong et al.^19^	China	Wuhan city	Tongji hospital	11 Jan 2020–5 Feb 2020	42	49.5 (14.1)	59.5%	NA
Arentz et al.^20^	US	Washington State	Evergreen Hospital	20 Feb 2020–5 Mar 2020	21	NA	52.4%	71.4%
Huang et al.^21^	China	Wuhan city	Jin Yin-tan Hospital	16 Dec 2019–2 Jan 2020	41	49.3 (13.1)	73.2%	NA
Guan et al.^22^	China	30 provinces, autonomous regions, and municipalities in mainland China		11 Dec 2019–29 Jan 2020	1099	46.7 (17.1)	58.0%	15.7%
Zhao et al.^23^	China	Anhui province	Second Affiliated Hospital of Anhui Medical University and Suzhou Municipal Hospital	23 Jan 2020–5 Feb 2020	19	43.7 (23.2)	57.9%	0.0%
Xu et al.^24^	China	Zhejiang province	Seven hospitals	10 Jan 2020–26 Jan 2020	62	41.7 (15.2)	56.5%	NA
Chan et al.^25^	China	Guangdong province	The University of Hong Kong-Shenzhen Hospital	10 Jan 2020–15 Jan 2020	7	46.2 (22.5)	50.0%	NA
Chen et al.^26^	China	Wuhan city	Jin Yin-tan Hospital	1 Jan 2020–20 Jan 2020	99	55.5 (13.1)	67.7%	17.2%
Pung et al.^27^	Singapore	Singapore	Not reported	3 Feb 2020- 8 Feb 2020	17	42.3 (12.1)	41.2%	NA
Wang et al.^28^	China	Wuhan city	Zhongnan Hospital	1 Jan 2020–28 Jan 2020	138	55.3 (19.5)	54.3%	19.6%
Young et al.^29^	Singapore	Singapore	Four hospitals	23 Jan 2020–3 Feb 2020	18	NA	50.0%	0.0%
Chen et al.^30^	China	Wuhan city	Zhongnan Hospital	20 Jan 2020–31 Jan 2020	9	32.0 (12.2)	NA	0.0%
Huang et al.^31^	Taiwan	Taichung	Taichung Veterans General Hospital	NA	2	73.5 (0.5)	0.0%	NA
Cheng et al.^32^	Taiwan	Taoyuan	Taoyuan General Hospital	20 Jan 2020	1	55.0 (NA)	0.0%	NA
Holshue et al.^33^	US	Washington	Not reported	20 Jan 2020	1	35.0 (NA)	100.0%	NA
Wei et al.^34^	China	Beijing city, Hainan, Guangdong, Anhui, Shanghai, Zhejiang, and Guizhou province	Not reported	8 Dec 2019–6 Feb 2020	9	0.5 (0.8)	22.2%	0.0%
Bernard-Stoecklin et al.^35^	France	Bordeaux and Paris	Not reported	10 Jan 2020–24 Jan 2020	3	36.3 (10.1)	66.7%	NA
Shi et al.^36^	China	Wuhan city	Jin Yin-tan hospital and Union Hospital of Tongji Medical College	20 Dec 2019–23 Jan 2020	81	49.5 (11.0)	51.9%	3.7%
Zhu et al.^37^	China	Wuhan city	Jin Yin-tan Hospital	27 Dec 2019	3	47.3 (14.6)	66.7%	100.0%
Ghinai et al.^38^	US	Illinois State	Not reported	20 Jan 2020–24 Jan 2020	2	NA	50.0%	NA
Zhou et al.^39^	China	Wuhan city	Jin Yin-tan Hospital and Wuhan Pulmonary Hospital	29 Dec 2019–31 Jan 2020	191	56.3 (15.7)	62.3%	62.3%
Yang et al.^70^	China	Wuhan city	Wuhan Jin Yin-tan	24 Dec 2019–26 Jan 2020	52	59.7 (13.3)	67.3%	100.0%
Kim et al.^41^	South Korea	Seoul	Incheon Medical Center, Seoul National University Hospital, and Seoul National University Bundang Hospital	21 Feb 2020	1	35.0 (NA)	0.0%	NA
Okada et al.^42^	Thailand	Nonthaburi	Bamrasnaradura Infectious Disease Institute Hospital	8 Jan 2020–13 Jan 2020	2	NA	0.0%	0.0%
Arashiro et al.^43^	Diamond Princess cruise ship			9 Feb 2020	2	31.0 (14.2)	50.0%	0.0%
Lillie et al.^44^	UK	Newcastle and Hull	Castle Hill Hospital	30 Jan 2020	2	36.5 (19.1)	50.0%	NA
Tian et al.^45^	China	Wuhan city	Zhongnan Hospital	NA	2	78.5 (19.5)	50.0%	NA
Haveri et al.^46^	Finland	Rovaniemi	Lapland Central Hospital	29 Jan 2020	1	NA	0.0%	NA
Nicastri et al.^47^	Italy	Rome	Lazzaro Spallanzani National Institute for Infectious Diseases	6 Feb 2020	1	NA	100.0%	NA
Cuong et al.^48^	Vietnam	Hanoi	Thanh Hoa General Hospital		1	25.0 (NA)	0.0%	NA
Spiteri et al.^49^	European region	Germany, France, Italy, Spain, Finland, Sweden, Belgium, Russia	Not reported	24 Jan 2020–21 Feb 2020	38	41.7 (NA)	65.8%	NA
Rothe et al.^50^	Germany	Munich		26 Jan 2020–28 Jan 2020	4	NA	NA	0.0%
Tong et al.^51^	China	Zhejiang Province	Not reported	19 Jan 2020–30 Jan 2020	7	31.1 (12.2)	42.9%	NA
Bai et al.^82^	China	Anyang city	Fifth People’s Hospital of Anyang	26 Jan 2020–28 Jan 2020	5	NA	0.0%	40.0%
Yu et al.^53^	China	Shanghai city	Not reported	22 Jan 2020–23 Jan 2020	4	76.5 (25.1)	50.0%	NA
Li et al.^84^	China	Zhejiang Province	Not reported	6 Feb 2020–9 Feb 2020	4	44.8 (27.4)	25.0%	NA
Tang et al.^55^	China	Zhejiang Province	Not reported	1 Feb 2020	1	10.0 (NA)	100.0%	NA
Kam et al.^56^	Singapore	Singapore	KK Women’s and Children’s Hospital	3 Feb 2020	1	0.5 (NA)	100.0%	NA
Zhou et al.^57^	China	Wuhan city	Tongji Hospital	16 Jan 2020–30 Jan 2020	62	52.8 (12.2)	62.9%	NA
Zhao et al.^58^	China	Hunan Province	Four hospitals	NA	101	44.4 (12.3)	55.4%	13.9%
Cheng et al.^59^	China	Shanghai city	Ruijin Hospital	19 Jan 2020–6 Feb 2020	11	50.4 (15.5)	72.7%	NA
Chung et al.^60^	China	Guangdong, Jiangxi, and Shandong Provinces	Three hospitals	18 Jan 2020–27 Jan 2020	21	51.0 (14.0)	61.9%	NA
Liu et al.^61^	China	Hubei province	Nine hospital	30 Dec 2019–24 Jan 2020	137	55.0 (16.0)	44.5%	NA
Chang et al.^62^	China	Beijing city	Three hospitals	16 Jan 2020–29 Jan 2020	13	38.7 (11.6)	76.9%	NA
COVID-19 National Incident Room Surveillance Team^63^	Australia	National-wide	Not reported	20 Jan 2020–14 Mar 2020	295	45.9 (17.4)	50.8%	NA
Pan et al.^64^	China	Wuhan city	Union Hospital	12 Jan 2020–6 Feb 2020	21	40.0 (9.0)	28.6%	0.0%
Wang et al.^65^	China	Wuhan city	Tongji Hospital	2 Feb 2020	1	0.0 (NA)	0.0%	NA
Bastola et al.^66^	Nepal	Kathmandu	Sukraraj Tropical and Infectious Disease Hospital	14 Jan 2020	1	32.0 (NA)	0.0%	NA
Qiu et al.^67^	China	Zhejiang Province	Three hospitals	17 Jan 2020–1 Mar 2020	36	8.3 (3.5)	63.9%	0.0%
Zhang et al.^98^	China	Wuhan city	No. 7 Hospital of Wuhan	16 Jan 2020–3 Feb 2020	140	0.0 (0.0)	50.7%	41.4%
Ye et al.^69^	China	Wuhan city	Zhongnan Hospital	8 Jan 2020–10 Feb 2020	5	32.4 (5.7)	40.0%	NA
Liu et al.^70^	China	Shenzhen	Shenzhen Third People’s Hospital	21 Jan 2020	12	52.8 (18.6)	66.7%	41.7%
Chen et al.^29^	China	Wuhan city	Tongji Hospital	13 Jan 2020–12 Feb 2020	274	58.7 (19.4)	62.4%	71.5%
Guan et al.^72^	China	31 province/autonomous regions/provincial municipalities	575 hospitals	11 Dec 2019–31 Jan 2020	1590	48.9 (16.3)	56.9%	16.0%
Wong et al.^73^	China	Hong Kong	Queen Mary Hospital, Pamela Youde Nethersole Eastern Hospital, Queen Elizabeth Hospital, and Ruttonjee Hospital	1 Jan 2020–5 Mar 2020	64	56.0 (19.0)	40.6%	NA
Xu et al.^74^	China	Changzhou	Third Hospital of Changzhou	23 Jan 2020–18 Feb 2020	51	42.3 (20.8)	49.0%	0.0%
Shen et al.^75^	China	Shenzhen	Shenzhen Third People's Hospital	20 Jan 2020–25 Mar 2020	5	54.0 (15.2)	60.0%	100.0%
Kimball et al.^76^	US	Washington State	Not reported	13 Mar 2020	23	80.7 (8.4)	30.4%	NA
Centers for Disease Control and Prevention^77^	US	49 states, district of Columbia, and 3 US territories	Not reported	12 Feb 2020–16 Mar 2020	4226	NA	NA	NA
Wu et al.^78^	China	Jiangsu Province	Three hospitals	22 Jan 2020–14 Feb 2020	80	46.1 (15.4)	48.8%	3.8%
Yang et al.^79^	China	Wenzhou city	Three hospitals	17 Jan 2020–10 Feb 2020	149	45.1 (13.4)	54.4%	NA
Zhu et al.^80^	China	Wuhan city	Tongji Hospital	4 Dec 2019	1	52.0 (NA)	100.0%	NA
Zhu et al.^81^	China	Hefei	Affiliated Hospital of University of Science and Technology of China	24 Jan 2020–20 Feb 2020	32	44.3 (13.2)	46.9%	NA
Wu et al.^82^	China	Wuhan city	Jinyintan Hospital	25 Dec 219–26 Jan 2020	201	51.3 (12.7)	63.7%	41.8%
Wang et al.^83^	China	Shanghai	Shanghai Public Health Clinical Center	21 Jan 2020–24 Jan 2020	4	44.3 (22.3)	75.0%	25.0%
Wang et al.^84^	China	Shenzhen	Shenzhen Third People's Hospital	11 Jan 2020–29 Feb 2020	55	39.9 (21.6)	40.0%	3.6%
Wan et al.^85^	China	Chongqing	Chongqing University Three Gorges Hospital	23 Jan 2020–8 Feb 2020	135	46.0 (14.2)	53.3%	29.6%
Tian et al.^86^	China	Beijing	57 Hospitals	20 Jan 2020–10 Feb 2020	262	45.9 (20.8)	48.5%	17.6%
Sun et al.^87^	China	Wuhan city	Wuhan Children’s Hospital	24 Jan 2020–24 Feb 2020	8	6.8 (6.5)	75.0%	100.0%
Song et al.^88^	China	Shanghai	Shanghai Public Health Clinical Center	20 Jan 2020–27 Jan 2020	51	49.0 (16.0)	49.0%	NA
Hu et al.^89^	China	Nanjing, Jiangsu Province	Second Hospital of Nanjing	28 Jan 2020–9 Feb 2020	24	38.9 (22.6)	33.3%	0.0%
Qu et al.^90^	China	Huizhou	Huizhou Municipal Central Hospital	Jan 2020–Feb 2020	30	50.5 (22.6)	53.3%	10.0%
Qian et al.^91^	China	Zhejiang	Five hospitals	20 Jan 2020–11 Feb 2020	91	47.8 (15.4)	40.7%	9.9%
Mo et al.^92^	China	Wuhan city	Zhongnan Hospital	1 Jan 2020–5 Feb 2020	155	54.0 (18.0)	55.5%	59.4%
Liu et al.^93^	China	Wuhan city	Three hospitals	30 Dec 2019–15 Jan 2020	78	42.7 (18.1)	50.0%	10.3%
Liu et al.^94^	China	Hainan	Hainan General Hospital	1 Jan 2020–15 Feb 2020	56	52.1 (14.7)	55.4%	NA
Liu et al.^95^	China	Hangzhou	Xixi hospital	22 Jan 2020–11 Feb 2020	10	43.0 (10.4)	40.0%	NA
Liu et al.^127^	China	Wuhan City	Union Hospital	20 Jan 2020–10 Feb 2020	15	32.0 (5.0)	0.0%	NA
Guillen et al.^97^	Spain	Not reported	Not reported	28 Feb 2020	1	50.0 (NA)	100.0%	NA
Dong et al.^98^	China	Wuhan City	Zhongnan Hospital of Wuhan University, Wuhan No.7 Hospital and Wuhan Children’s Hospital	NA	11	36.6 (21.5)	45.5%	9.1%
Fan et al.^99^	China	Not reported	Not reported	24 Jan 2020–26 Jan 2020	1	31.5 (3.5)	0.0%	NA
Chen et al.^100^	China	Wuhan City	Renmin hospital of Wuhan University	30 Jan 2020–23 Feb 2020	17	29.4 (2.9)	0.0%	NA
Chen et al.^101^	China	Wuhan City	Zhongnan Hospital of Wuhan University	2 Jan 2020	2	NA	0.0%	NA
Chen et al.^102^	China	Shanghai	Shanghai Public Health Clinical Center	20 Jan 2020–6 Feb 2020	249	50.3 (20.9)	50.6%	10.0%
Ding et al.^103^	China	Wuhan City	Tongji Hospital	NA	5	50.2 (9.8)	40.0%	NA
Kong et al.^104^	Korea	Not reported	Not reported	20 Jan 2020–14 Feb 2020	28	42.6 (NA)	53.6%	NA
Li et al.^105^	China	Zhengzhou City	Not reported	5 Feb 2020	2	4.0 (0.0)	50.0%	NA
Ai et al.^106^	China	Shanghai	Not reported	20 Jan 2020	1	56.0 (NA)	0.0%	NA

*COVID-19* Coronavirus Disease 2019, *US* The United States, *UK* The United Kingdom, *SD *standard deviation, *NA* not available.

Of those 90 eligible studies, 11,028 COVID-19 patients were identified and included in the systematic review. More than half of patients (6336, 57.5%) were from mainland China. The pooled mean age was 45.8 (95% CI 38.6–52.5) years and 49.3% (pooled 95% CI 45.6–53.0%) of them were male.

**Figure 2:**
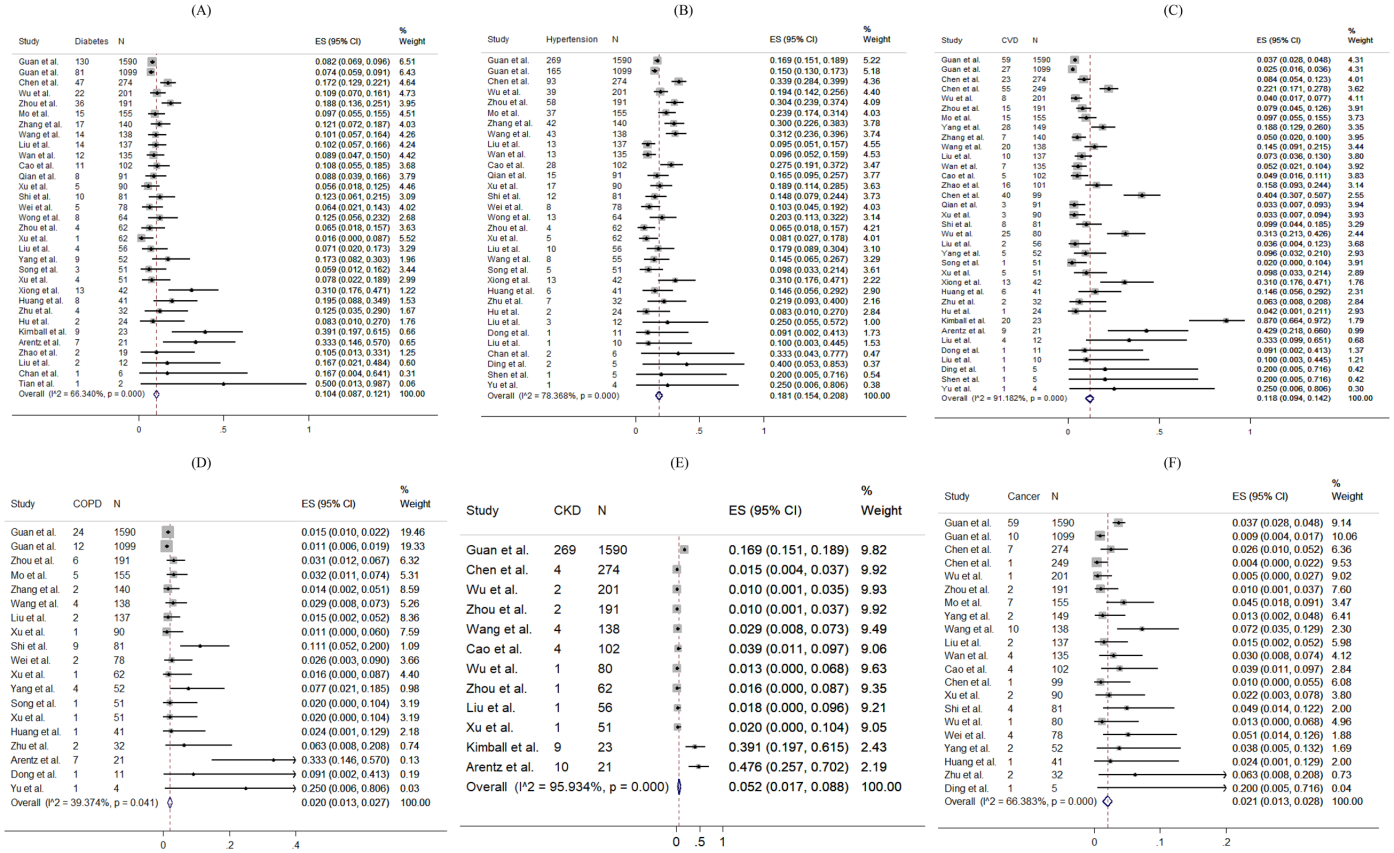
Random-effects meta-analytic estimates for comorbidities. (**A**) Diabetes mellitus, (**B**) Hypertension, (**C**) Cardiovascular disease, (**D**) Chronic obstructive pulmonary disease, (**E**) Chronic kidney disease, (**F**) Cancer.

For specific comorbidity status, the most prevalent comorbidity was hypertension (18.1%, 95% CI 15.4–20.8%), followed by cardiovascular disease (11.8%, 95% CI 9.4–14.2%) and diabetes (10.4%, 95% CI 8.7–12.1%). The pooled prevalence (95% CI) of COPD, chronic kidney disease, liver disease and cancer were 2.0% (1.3–2.7%), 5.2% (1.7–8.8%), 2.5% (1.7–3.4%) and 2.1% (1.3–2.8%) respectively. Moderate to substantial heterogeneity between reviewed studies were found, with I^2^ statistics ranging from 39.4 to 95.9% (*p* values between < 0.001–0.041), except for liver disease (I^2^ statistics: 1.7%, *p* = 0.433). Detailed results for comorbidity status are displayed in Fig. 2.

**Figure 3:**
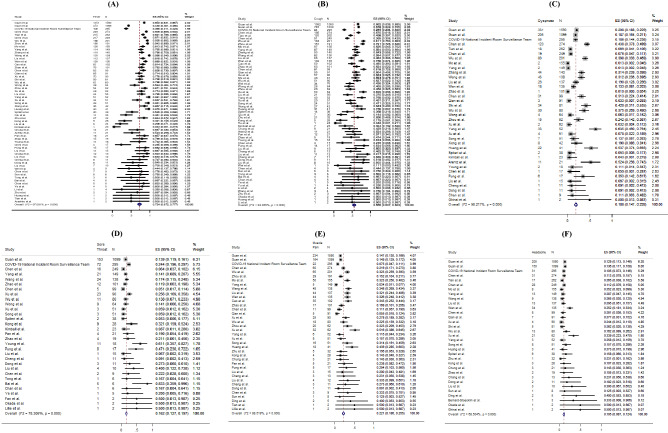
Random-effects meta-analytic estimates for presenting symptoms. (**A**) Fever, (**B**) Cough, (**C**) Dyspnoea, (**D**) Sore throat, (**E**) Muscle pain, (**F**) Headache.

Regarding the symptoms presented at hospital admission, the most frequent symptoms were fever (pooled prevalence: 72.4%, 95% CI 67.2–77.7%) and cough (pooled prevalence: 55.5%, 95% CI 50.7–60.3%). Sore throat (pooled prevalence: 16.2%, 95% CI 12.7–19.7%), dyspnoea (pooled prevalence: 18.8%, 95% CI 14.7–22.8%) and muscle pain (pooled prevalence: 22.1%, 95% CI 18.6–25.5%) were also common symptoms found in COVID-19 patients, but headache (pooled prevalence: 10.5%, 95% CI 8.7–12.4%), diarrhoea (pooled prevalence: 7.9%, 95% CI 6.3–9.6%), rhinorrhoea (pooled prevalence: 9.2%, 95% CI 5.6–12.8%) were less common. However, none of the included papers reported prevalence of anosmia and ageusia. The I^2^ statistics varied from 68.5 to 97.1% (all *p* values < 0.001), indicating a high heterogeneity exists across studies. Figure 3 shows the pooled proportion of symptoms of patients presented at hospital.

**Figure 4:**
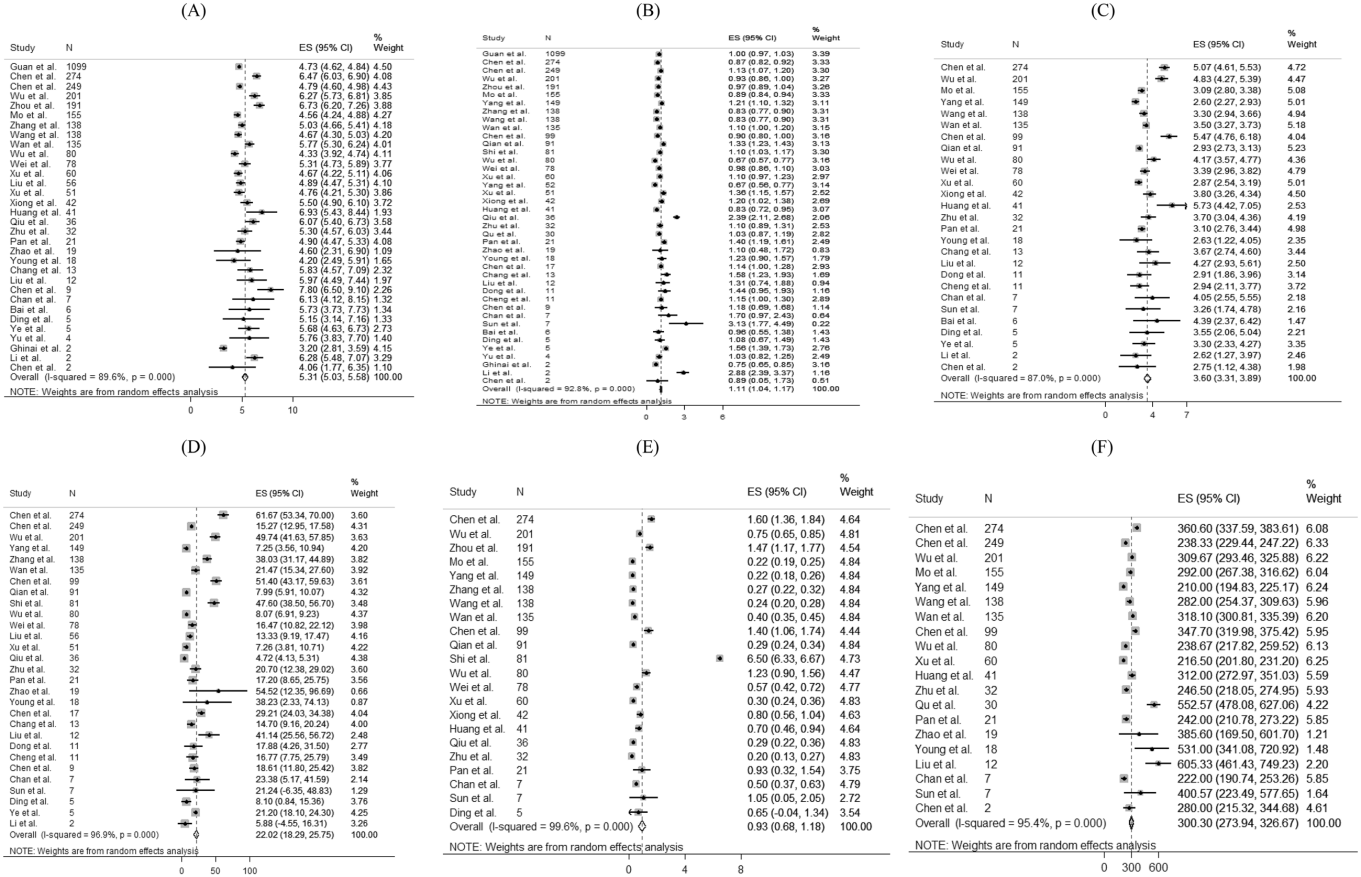
Random-effects meta-analytic estimates for laboratory parameters. (**A**) White blood cell, (**B**) Lymphocyte, (**C**) Neutrophil, (**D**) C-creative protein, (**E**) D-dimer, (**F**) Lactate dehydrogenase.

For laboratory parameters, white blood cell (pooled mean: 5.31 × 10^9^/L, 95% CI 5.03–5.58 × 10^9^/L), neutrophil (pooled mean: 3.60 × 10^9^/L, 95% CI 3.31–3.89 × 10^9^/L), lymphocyte (pooled mean: 1.11 × 10^9^/L, 95% CI 1.04–1.17 × 10^9^/L), platelet count (pooled mean: 179.5 U/L, 95% CI 172.6–186.3 U/L), aspartate aminotransferase (pooled mean: 30.3 U/L, 95% CI 27.9–32.7 U/L), alanine aminotransferase (pooled mean: 27.0 U/L, 95% CI 24.4–29.6 U/L) and C-reactive protein (CRP) (pooled mean: 22.0 mg/L, 95% CI 18.3–25.8 mg/L) and D-dimer (0.93 mg/L, 95% CI 0.68–1.18 mg/L) were the common laboratory test taken for COVID-19 patients. Above results and other clinical factors are depicted in Fig. 4. Same with the comorbidity status and symptoms, high likelihood of heterogeneity was detected by I^2^ statistics for a majority of clinical parameters.

**Figure 5:**
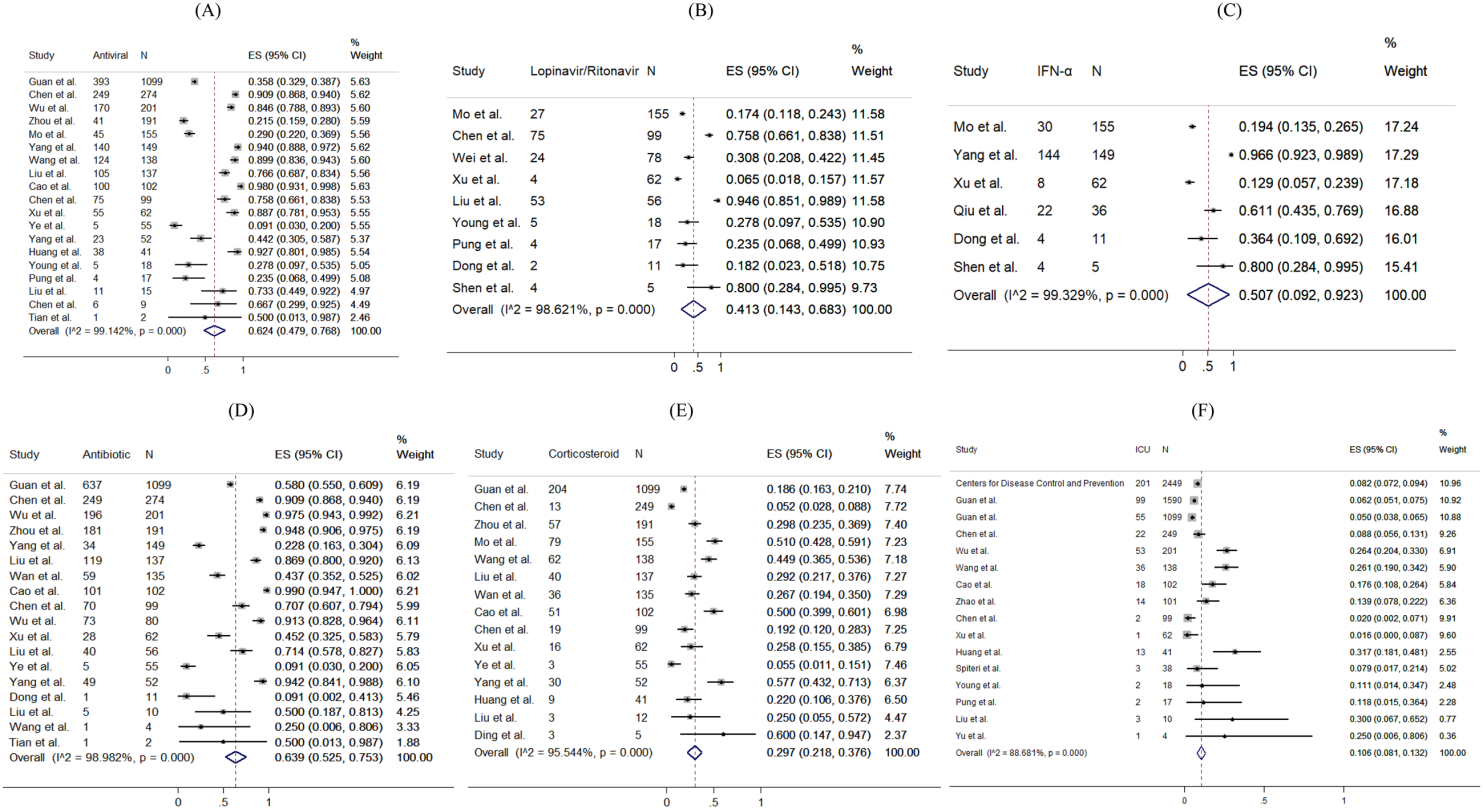
Random-effects meta-analytic estimates for pharmacological treatments and intensive unit care at hospital. (**A**) Antiviral or interferon drugs, (**B**) Lopinavir/Ritonavir, (**C**) Interferon alpha (IFN-α), (**D**) Antibiotic drugs, (**E**) Corticosteroid, (**F**) Admission to Intensive care unit.

Figure 5 presents the distribution of the pharmacological treatments received for COVID-19 patients. 10.6% of patients admitted to intensive care units (pooled 95% CI 8.1–13.2%). For drug treatment, 63.9% (pooled 95% CI 52.5–75.3%), 62.4% (pooled 95% CI 47.9–76.8%) and 29.7% (pooled 95% CI 21.8–37.6%) patients used antibiotics, antiviral, and corticosteroid, respectively. 41.3% (pooled 95% CI 14.3–68.3%) and 50.7% (pooled 95% CI 9.2–92.3%) reported using Lopinavir/Ritonavir and interferon-alpha as antiviral drug treatment, respectively. Among 14 studies reporting proportion of corticosteroid used, 7 studies (50%) specified the formulation of corticosteroid as systemic corticosteroid. The remaining one specified the use of methylprednisolone. No reviewed studies reported the proportion of patients receiving Ribavirin, Interferon-beta, or inotropes.

**Figure 6:**
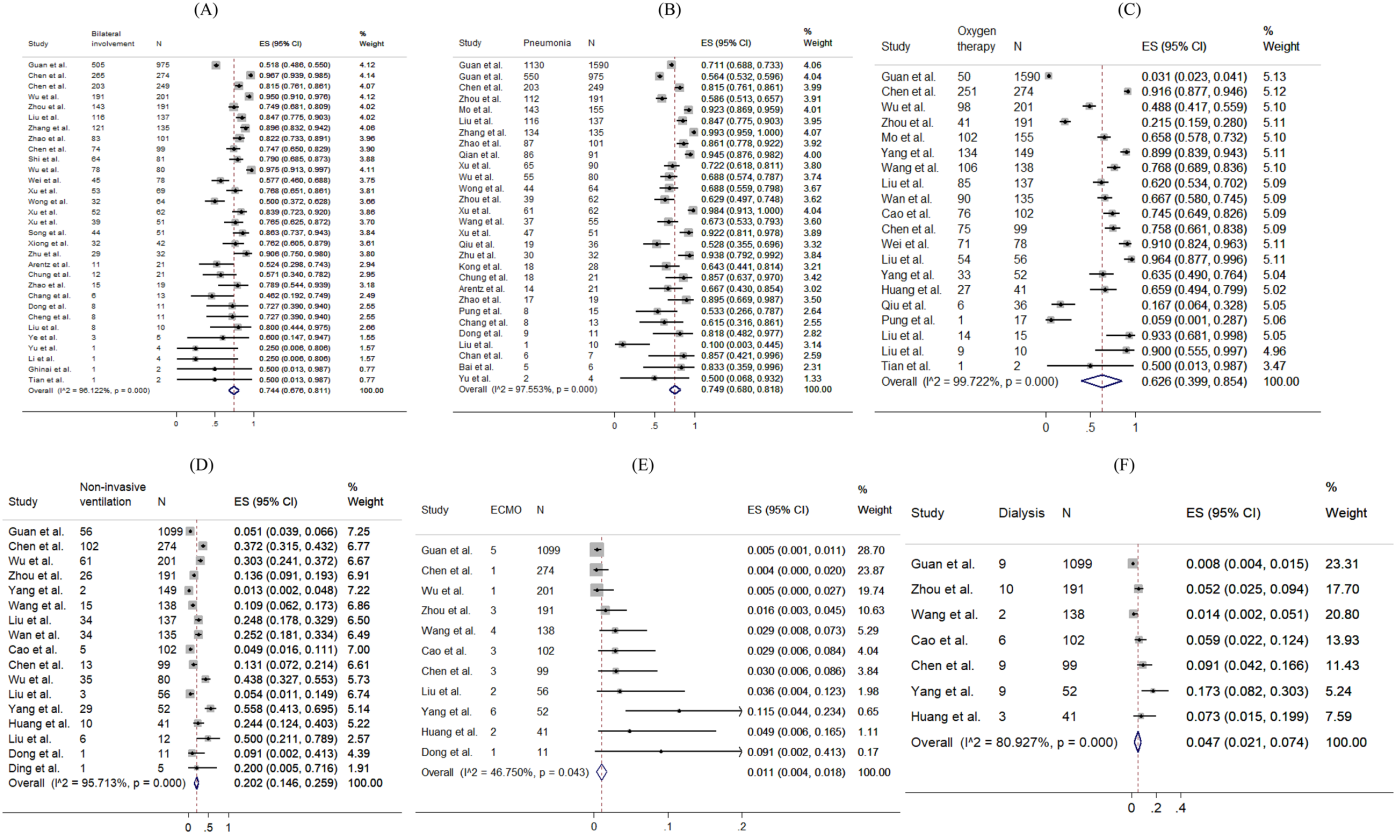
Random-effects meta-analytic estimates for radiological findings and non-pharmacological treatments at hospital. (**A**) Bilateral involvement, (**B**) Pneumonia, (**C**) Oxygen therapy, (**D**) Non-invasive ventilation, (**E**) Extracorporeal membrane oxygenation (ECMO), (**F**) Dialysis.

The prevalence of radiological outcomes and non-pharmacological treatments were presented in Fig. 6. Radiology findings detected chest X-ray abnormalities, with 74.4% (95% CI 67.6–81.1%) of patients with bilateral involvement and 74.9% (95% CI 68.0–81.8%) of patients with viral pneumonia. 62.6% (pooled 95% CI 39.9–85.4%), 20.2% (pooled 95% CI 14.6–25.9%), 15.3% (pooled 95% CI 11.0–19.7%), 1.1% (pooled 95% CI 0.4–1.8%) and 4.7% (pooled 95% CI 2.1–7.4%) took oxygen therapy, non-invasive ventilation, mechanical ventilation, ECMO and dialysis respectively.

The funnel plots and results Egger’s test of comorbidity status, symptoms presented, laboratory test and treatment were presented in eFigure 1–S5 in the Supplement. 63% (19/30) of the funnel plots (eFigure 1–S5) showed significance in the Egger’s test for asymmetry, suggesting the possibility of publication bias or small-study effects caused by clinical heterogeneity.

## Discussion

This meta-analysis reveals the condition of global medical community responding to COVID-19 in the early phase. During the past 4 months, a new major epidemic focus of COVID-19, some without traceable origin, has been identified. Following its first identification in Wuhan, China, the virus has been rapidly spreading to Europe, North America, Asia, and the Middle East, in addition to African and Latin American countries. Three months since Wuhan CDC admitted that there was a cluster of unknown pneumonia cases related to Huanan Seafood Market and a new coronavirus was identified as the cause of the pneumonia^108^, as on 1 April, 2020, there have been 858,371 persons confirmed infected with COVID-19, affecting 202 countries and territories around the world. Although this rapid review is limited by the domination of reports from patients in China, and the patient population is of relative male dominance reflecting the gender imbalance of the Chinese population^109^, it provides essential information.

In this review, the pooled mean age was 45.8 years. Similar to the MERS-CoV pandemic^110^, middle-aged adults were the at-risk group for COVID-19 infections in the initial phase, which was different from the H1N1 influenza pandemic where children and adolescents were more frequently affected^111^. Biological differences may affect the clinical presentations of infections; however, in this review, studies examining the asymptomatic COVID-19 infections or reporting any previous infections were not included. It is suggested that another systematic review should be conducted to compare the age-specific incidence rates between the pre-pandemic and post-pandemic periods, so as to understand the pattern and spread of the disease, and tailor specific strategies in infection control.

Both sexes exhibited clinical presentations similar in symptomatology and frequency to those noted in other severe acute respiratory infections, namely influenza A H1N1^112^ and SARS^113,114^. These generally included fever, new onset or exacerbation of cough, breathing difficulty, sore throat and muscle pain. Among critically ill patients usually presented with dyspnoea and chest tightness^22,29,39,72^, 141 (4.6%) of them with persistent or progressive hypoxia resulted in the requirement of intubation and mechanical ventilation^115^, while 194 (6.4%) of them required non-invasive ventilation, yielding a total of 11% of patients requiring ventilatory support, which was similar to SARS^116^.

The major comorbidities identified in this review included hypertension, cardiovascular diseases and diabetes mellitus. Meanwhile, the percentages of patients with chronic renal diseases and cancer were relatively low. These chronic conditions influencing the severity of COVID-19 had also been noted to have similar effects in other respiratory illnesses such as SARS, MERS-CoV and influenza^117,118^. Higher mortality had been observed among older patients and those with comorbidities.

Early diagnosis of COVID-19 was based on recognition of epidemiological linkages; the presence of typical clinical, laboratory, and radiographic features; and the exclusion of other respiratory pathogens. The case definition had initially been narrow, but was gradually broadened to allow for the detection of more cases, as milder cases and those without epidemiological links to Wuhan or other known cases had been identified^119,120^. Laboratory investigations among COVID-19 patients did not reveal specific characteristics—lymphopenia and elevated inflammatory markers such as CRP are some of the most common haematological and biochemical abnormalities, which had also been noticed in SARS^121^. None of these features were specific to COVID-19. Therefore, diagnosis should be confirmed by SARS-CoV–2 specific microbiological and serological studies, although initial management will continue to be based on a clinical and epidemiological assessment of the likelihood of a COVID-19 infection.

Radiology imaging often plays an important role in evaluating patients with acute respiratory distress; however, in this review, radiological findings of SARS-CoV-2 pneumonia were non-specific. Despite chest radiograph usually revealed bilateral involvement and Computed Tomography usually showed bilateral multiple ground-glass opacities or consolidation, there were also patients with normal chest radiograph, implying that chest radiograph might not have high specificity to rule out pneumonia in COVID-19.

Limited clinical data were available for asymptomatic COVID-19 infected persons. Nevertheless, asymptomatic infection could be unknowingly contagious^122^. From some of the official figures, 6.4% of 150 non-travel-related COVID-19 infections in Singapore^123^, 39.9% of cases from the Diamond Princess cruise ship in Japan^124^, and up to 78% of cases in China as extracted on April 1st, 2020, were found to be asymptomatic^122^. 76% (68/90) studies based on hospital setting which provided care and disease management to symptomatic patients had limited number of asymptomatic cases of COVID-19 infection. This review calls for further studies about clinical data of asymptomatic cases. Asymptomatic infection intensifies the challenges of isolation measures. More global reports are crucially needed to give a better picture of the spectrum of presentations among all COVID-19 infected persons. Also, public health policies including social and physical distancing, monitoring and surveillance, as well as contact tracing, are necessary to reduce the spread of COVID-19.

Concerning potential treatment regime, 62.4% of patients received antivirals or interferons (including oseltamivir, lopinavir-ritonavir, interferon alfa), while 63.9% received antibiotics (such as moxifloxacin, and ceftriaxone). In this review, around one-third of patients were given steroid, suggestive as an adjunct to IFN, or sepsis management. Interferon and antiviral agents such as ribavirin, and lopinavir-ritonavir were used during SARS, and the initial uncontrolled reports then noted resolution of fever and improvement in oxygenation and radiographic appearance^113,125,126^, without further evidence on its effectiveness. At the time of manuscript preparation, there has been no clear evidence guiding the use of antivirals^127^. Further research is needed to inform clinicians of the appropriate use of antivirals for specific groups of infected patients.

Limitations of this meta-analysis should be considered. First, a high statistical heterogeneity was found, which could be related to the highly varied sample sizes (9 to 4226 patients) and study designs. Second, variations of follow-up period may miss the event leading to heterogeneity. In fact, some patients were still hospitalized in the included studies. Third, since only a few studies had compared the comorbidities of severe and non-severe patients, sensitivity analysis and subgroup analysis were not conducted. Fourthly, the frequency and severity of signs and symptoms reported in included studies, primarily based on hospitalized COVID-19 patients were over-estimated. Moreover, different cutoffs for abnormal laboratory findings were applied across countries, and counties within the same countries. Lastly, this meta-analysis reviewed only a limited number of reports written in English, with a predominant patient population from China. This review is expected to inform clinicians of the epidemiology of COVID-19 at this early stage. A recent report estimated the number of confirmed cases in China could reach as high as 232,000 (95% CI 161,000, 359,000) with the case definition adopted in 5th Edition. In this connection, further evidence on the epidemiology is in imminent need.

## Supplementary information

Supplementary Figure 1.

Supplementary Figure 2.

Supplementary Figure 3.

Supplementary Figure 4.

Supplementary Figure 5.

Supplementary Material 6.
