# Supplementary material for: Clinical presentations, laboratory and radiological findings, and treatments for 11,028 COVID-19 patients: a systematic review and meta-analysis
Source: Sci Rep. 2020 Nov 13;10:19765. doi: 10.1038/s41598-020-74988-9 (PMC7666204; doi:10.1038/s41598-020-74988-9)

**Clinical presentations, laboratory and radiological findings, and treatments for 11,028 COVID-19 patients: a systematic review and meta-analysis**

**Carlos K.H. Wong^1,2^***, BSc (Hons), MPhil, PhD.

**Janet Y. Wong^3^***, RN, PhD.

**Eric H.M. Tang^1^**, BSc (Hons)

**Chi Ho Au^1^**, BSc (Hons)

**Abraham K. Wai^4^**, MBChB, MSc, JD, MBA, FHKAM (Emergency Medicine).

*Contributed equally to this manuscript

^1^Department of Family Medicine and Primary Care, Li Ka Shing Faculty of Medicine, The University of Hong Kong, Hong Kong, China

^2^Department of Pharmacology and Pharmacy, Li Ka Shing Faculty of Medicine, The University of Hong Kong, Hong Kong, China

^3^School of Nursing, Li Ka Shing Faculty of Medicine, The University of Hong Kong, Hong Kong, China

^4^Emergency Medicine Unit, Li Ka Shing Faculty of Medicine, The University of Hong Kong, Hong Kong, China

**Corresponding Author**: Abraham K. Wai, Emergency Medicine Unit, Li Ka Shing Faculty of Medicine, The University of Hong Kong, Hong Kong SAR, China. Address: Room 514, William MW Mong Block, Faculty of Medicine Building, 21 Sassoon Road, Pokfulam, Hong Kong SAR, China. Tel: (+852) 3917-9859 Fax: (+852) 2816-2293. Email: awai@hku.hk

Supplementary Figure 3. Funnel plots for laboratory parameters. (A) White blood cell, (B) Lymphocyte, (C) Neutrophil, (D) C-reactive protein, (E) D-dimer, (F) Lactate dehydrogenase


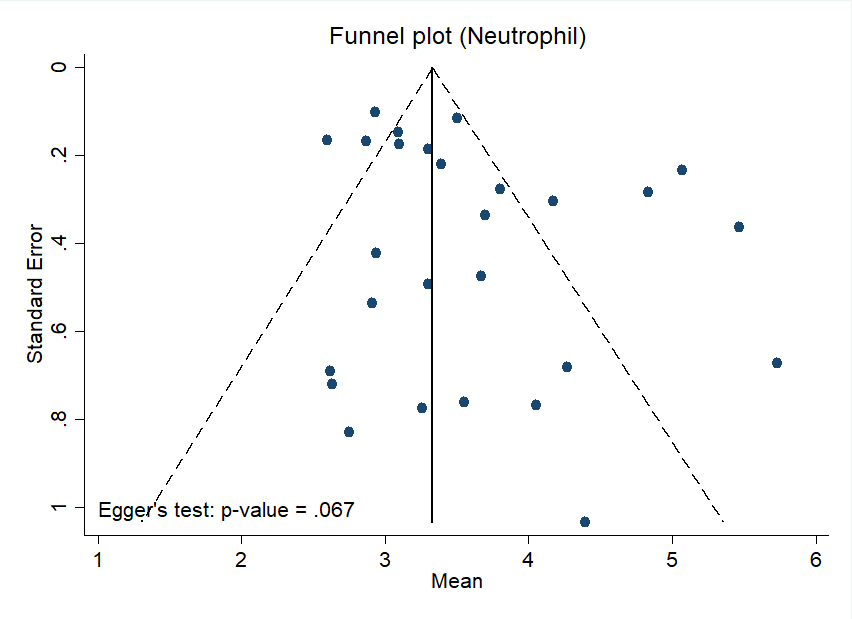
 (A) (B) (C)


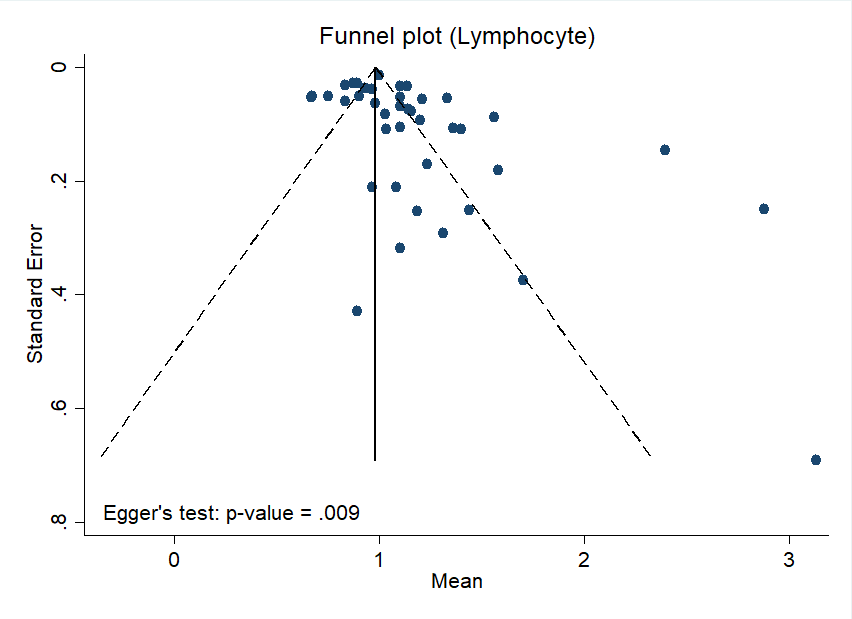

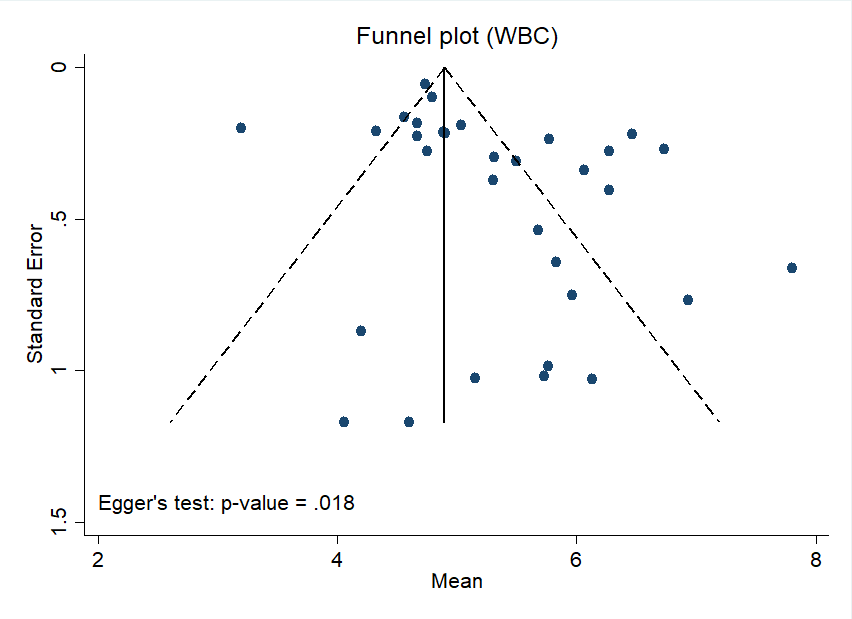


(D) (E) (F)


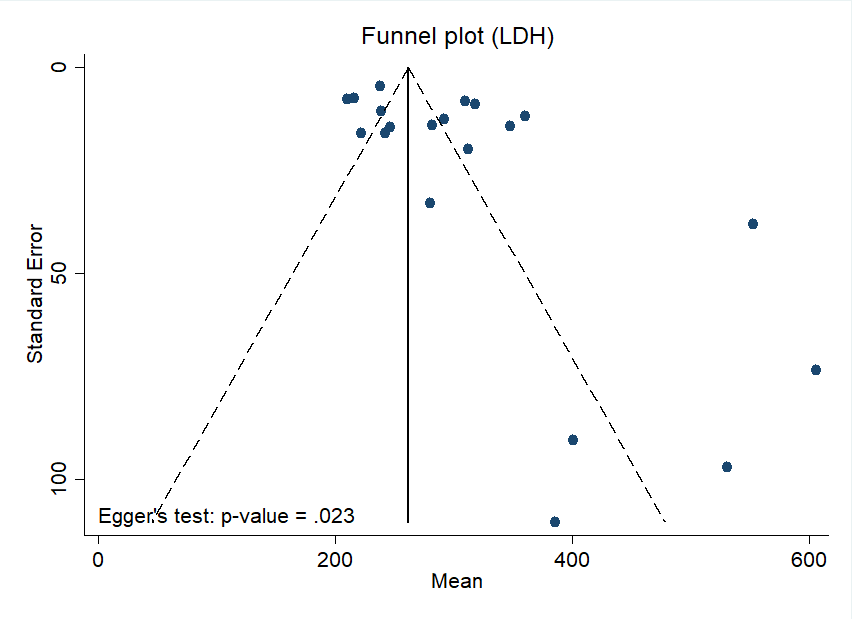

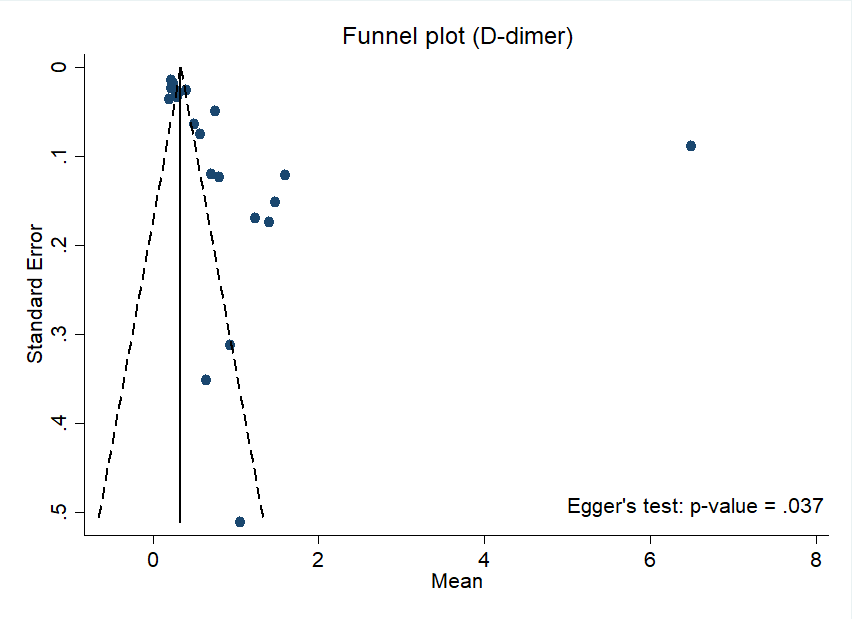

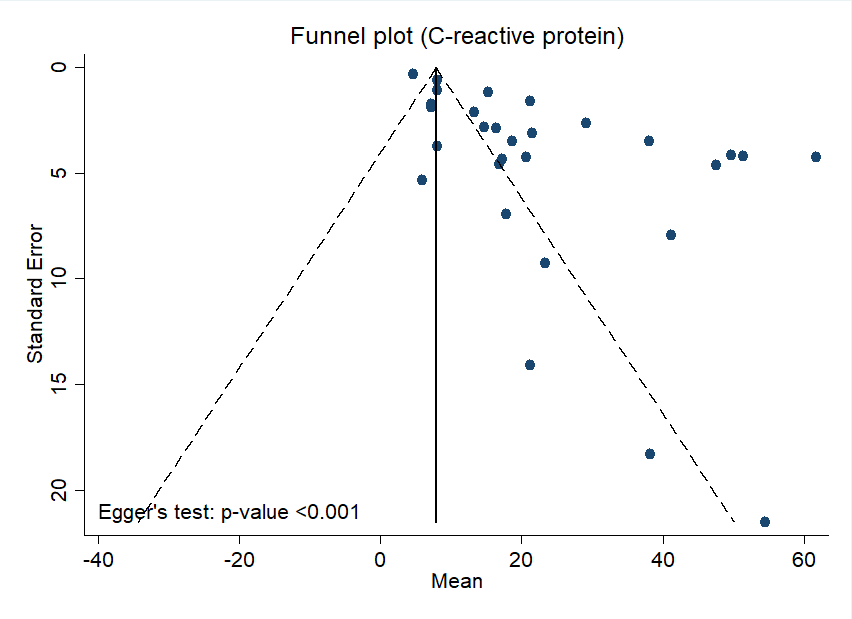

Supplement: Supplementary file 3 — Supplementary Figure 3. [file 41598_2020_74988_MOESM3_ESM.docx]
