# Supplementary material for: Clinical presentations, laboratory and radiological findings, and treatments for 11,028 COVID-19 patients: a systematic review and meta-analysis
Source: Sci Rep. 2020 Nov 13;10:19765. doi: 10.1038/s41598-020-74988-9 (PMC7666204; doi:10.1038/s41598-020-74988-9)
Supplement: Supplementary file 6 — Supplementary Material 6. [file 41598_2020_74988_MOESM6_ESM.docx]

**Clinical presentations, laboratory and radiological findings, and treatments for 11,028 COVID-19 patients: a systematic review and meta-analysis**

**Carlos K.H. Wong^1,2^***, BSc (Hons), MPhil, PhD.

**Janet Y. Wong^3^***, RN, PhD.

**Eric H.M. Tang^1^**, BSc (Hons)

**Chi Ho Au^1^**, BSc (Hons)

**Abraham K. Wai^4^**, MBChB, MSc, JD, MBA, FHKAM (Emergency Medicine).

*Contributed equally to this manuscript

^1^Department of Family Medicine and Primary Care, Li Ka Shing Faculty of Medicine, The University of Hong Kong, Hong Kong, China

^2^Department of Pharmacology and Pharmacy, Li Ka Shing Faculty of Medicine, The University of Hong Kong, Hong Kong, China

^3^School of Nursing, Li Ka Shing Faculty of Medicine, The University of Hong Kong, Hong Kong, China

^4^Emergency Medicine Unit, Li Ka Shing Faculty of Medicine, The University of Hong Kong, Hong Kong, China

**Corresponding Author**: Abraham K. Wai, Emergency Medicine Unit, Li Ka Shing Faculty of Medicine, The University of Hong Kong, Hong Kong SAR, China. Address: Room 514, William MW Mong Block, Faculty of Medicine Building, 21 Sassoon Road, Pokfulam, Hong Kong SAR, China. Tel: (+852) 3917-9859 Fax: (+852) 2816-2293. Email: awai@hku.hk

**Appendix 1**

Ovid MEDLINE(R) 1946 to March Week 3 2020,

HKU Journals@Ovid,

Journals@Ovid Full Text March 27, 2020

Embase Classic+Embase 1947 to 2020 March 27

| **Step** | **Keyword/Strategy** |
| --- | --- |
| **1** | **COVID** |
| **2** | **SARS-CoV-2** |
| **3** | **novel pneumonia** |
| **4** | **epidemiology** |
| **5** | **clinical characteristics** |
| **6** | **outcome** |
| **7** | **1 or 2 or 3** |
| **8** | **4 or 5 or 6** |
| **9** | **7 and 8** |
| **10** | **limit 9 to abstracts** |
| **11** | **limit 10 to yr="2020"** |
